# Separating the “Deed” From the “Done-To”: How Communicating With the Offender Can Change Victims’ Self-Concept

**DOI:** 10.1177/08862605221119725

**Published:** 2022-09-04

**Authors:** Diana Batchelor

**Affiliations:** 1 University of Oxford, UK

**Keywords:** criminology, mental health and violence, memory and trauma, sexual assault, child abuse

## Abstract

Some victims benefit from communication with offenders after a crime, at least some of the time, but gaps in the evidence about restorative justice practices make it hard for victims to decide whether to take part. This article examines whether and how specific components of victim–offender communication led to changes in victim self-concept. The question was addressed through thematic analysis of interview data from 40 victims of a range of crime types, including serious sexual and violent offences. Interviews were conducted before and after victims attempted communication with the offender. Participants described 10 routes to change in their sense of agency and of being a “good” person (moral self-image); some of these routes supported previous literature, others shed new light on old theories, or were previously undocumented. Together, these routes enabled victims to distance themselves from a “victim” identity, thereby mirroring the commonly cited restorative justice objective of separating the “deed from the doer,” to instead separate the “deed from the done-to.” To the extent possible given the nature of the study, cases of negative and absent changes are also discussed. In an area replete with theories but lacking in empirical research, this study contributes new evidence and a conceptual clarity that could be used to enhance future studies. Most importantly, it can help victims make informed decisions about communicating with the offender, help them identify and articulate their objectives, and manage their expectations.

I don’t understand why [the facilitators] think it’s going to be so good for me. . . I don’t think it’s like a one size fits all. . . If it was a 17-year-old boy who broke into a house, how is that the same as this? . . . I don’t know why they are so convinced either that it’s going to work. Where’s their evidence? Michelle^1^, participant interview

When Michelle reported the physical and sexual abuse she had experienced as a child, her father was arrested and convicted. After he was released from prison, Michelle considered talking to him about the abuse, so she sought out a local restorative justice organization to help. The facilitators contacted Michelle’s father who agreed to meet her, and while considering whether to proceed she raised some important issues, illustrated in the quotation above. She may not have reviewed the academic literature, but she rightly identified gaps in the evidence that could affect her decision. As she was aware and as I detail below, there is evidence that at least some victims benefit from communication with the offender at least some of the time. Yet, there is little evidence to help her predict how her experience would be affected by the nature of the offence, her relationship with the offender, or the fact that her father was willing to apologize but could not explain why he committed the abuse.

Restorative justice practices consist primarily, but not exclusively, of a variety forms of communication between the victim and the offender,^2^ intended to deliver such benefits as “repair” and “restoration” to victims, offenders and the community (Braithwaite, 1989; Eglash, 1977; Fattah, 1998; Zehr, 1990). Indeed, many decades of research have demonstrated a range of benefits for victims, for example, some victims are more satisfied by meetings with the offender than conventional justice processes (Shapland et al., 2007). Victims often feel less fearful or angry after communicating with the offender, and some victims blame themselves less for the offence (Armstrong, 2012; Beven et al., 2005; Bolívar, 2013; Hallam, 2015; Hoyle et al., 2002; Miller & Iovanni, 2013; Rugge & Scott, 2009; Sherman et al., 2005; Strang, 2002; Strang et al., 2006; Umbreit et al., 1994; Wemmers & Cyr, 2004, 2005). Many victims appreciate having control over the process (Dignan, 2004; Tamarit & Luque, 2016; Van Camp & Wemmers, 2013; Wemmers & Cyr, 2005), leading in at least some cases to an increased sense of generalized control and agency (Bolitho, 2015; Jacobsson et al., 2012; Pelikan, 2010). Some victims experience reduction in symptoms of trauma (Angel et al., 2014; Lloyd & Borrill, 2020). A recent systematic review identified 35 studies which documented psychological change as a result of restorative justice interventions (Nascimento et al., 2022), ranging from changes in perceptions of the offender, to closure and recovery.

Despite this burgeoning field of research, the gaps in evidence mean it is still very difficult for victims such as Michelle—deciding whether to communicate with the offender—to know whether any of these potential benefits will apply in their specific circumstances. There are four main areas in which we lack evidence, and while the current study of course cannot address them all, I describe each gap here in more depth and indicate how the current study contributes.

First, because restorative justice has been used as an umbrella term, studies may assess the impact of a variety of different interventions yet refer to them all as restorative justice (Daly, 2016). This means it is hard for victims to decide whether what they are being offered will lead to the same benefits described in the literature. Not only might the evidence about “restorative justice” refer to practices as far removed from victim–offender communication as community service or prison-based victim-awareness courses, but the form and content of victim–offender communication also varies widely. While studies usually do differentiate between direct and indirect communication (e.g., Bolívar, 2013), the specific components of victim–offender communication are rarely disaggregated. In this study, I distinguish between individual components of communication with the offender (such as receiving an apology or discussing the impact of the offence), and I identify the intermediate psychological changes prompted by each component.

Second, very few studies have investigated the potential negative effects of participation, despite evidence of their existence at least some of the time (Choi et al., 2012; Morris et al., 2009; Rugge & Scott, 2009). While this could be because negative effects are extremely rare, it could also be because victims who have bad experiences are reluctant to participate in research interviews, or because practitioners are reticent to refer cases for research which might damage their reputation. Most concerningly, the absence of research on negative effects may also result from a definition of restorative justice as a paradigm, or set of values (Von Hirsch et al., 2003), rather than a specific practice. Such definitions effectively predetermine the outcomes of restorative justice, resulting in a circular argument. To be “restorative,” the argument goes, the victim must feel they achieved a sense of “restoration.” This means that a process lacking in victim restoration can never be a failure of restorative justice, it could only ever mean that the process was insufficiently “restorative.” In this study, I attempt to avoid some of these pitfalls by receiving referrals and seeking victims’ consent prior to the restorative justice process, and interviewing them both before and after the process wherever possible. This avoids the phenomenon of victims agreeing or facilitators referring only when cases are positive or “sufficiently restorative.”

Third, small sample sizes in much research on victims’ experiences of restorative justice practices have made it difficult to investigate factors which moderate the extent to which victims benefit. This gap is an almost unavoidable result of the many ethical and practical difficulties of conducting research with victims, and it is not one I am able to redress in the current study. However, this study makes a small contribution to mapping the effects for victims across a *range* of crime types and circumstances, by including serious and complex crimes to supplement research which more frequently includes only minor and nonsexual crimes.

Fourth, few studies have made explicit attempts to understand *how* communication with the offender benefits victims. Again, this absence in the literature is understandable given the type of data it is possible to collect from victims participating in restorative justice schemes. A comprehensive test of the mechanisms involved would require analysis of large-scale quantitative datasets, preferably with randomized control groups, which are largely incompatible with the individualized, usually small-scale services offered by most restorative justice organizations. Nonetheless, some authors have attempted to delve into the processes that might explain how victims benefit (Bolitho, 2017; Strang et al., 2006; Wemmers & Cyr, 2005) and, given its importance, it is not an exercise that should be abandoned simply because it is too difficult. In this study, therefore, I ask not only whether victims achieved their goal of feeling better, but whether and how victims’ felt the process led to an *intermediate* psychological change: change in self-concept. In the next section, I explain the rationale for this approach.

## Why Self-Concept?

In the study described below, victims frequently and spontaneously mentioned aspects of self-concept, having been asked open-ended questions about changes they experienced through communicating with the offender. The primary rationale for developing this theme, therefore, was that it was clearly of importance to the victims in this study.

Existing restorative justice literature implies that communication with the offender changes victims’ self-concept, but rarely explicitly uses self-concept as a category of psychological change. While changes in self-concept are a relatively common means of explaining *offenders*’ experiences of restorative justice schemes (e.g., Armour & Sliva, 2018), the few existing theories regarding changes in victim self-concept focus on specific routes to change. Procedural justice theorists propose that experiencing a just process enhances victims’ sense of agency and makes them feel valued by society (Tyler, 1989; Tyler & Blader, 2003). Sherman et al. (2005) propose that communicating with the offender challenges the extent to which the victim believes they were the cause of the crime, thereby addressing self-blame and shame. Multiple authors suggest that victims become less afraid of the offender through meeting them (Umbreit et al., 1994; Wallis, 2014; Walters, 2015), with some suggesting that humanization of the offender results in a feeling of relative strength and agency for victims (Starbuck, 2016). While each of these theories may partially explain how a restorative justice process leads to changes in self-concept, there are likely to be a range of such individual routes, which have not to date been comprehensively mapped. Using self-concept as a distinct subset of intermediate psychological changes enables the current study to delve into the nature of these routes and identify those missing from the literature to date.

Self-concept is also a useful boundaried subset of potential intermediate psychological changes because self-concept and its subdimensions are well documented in other domains. There has been much research on distinguishable but sometimes overlapping aspects of self-concept (e.g., self-image, self-esteem, self-efficacy, self-evaluation, self-knowledge, self-worth), dominated by a reasonably well-accepted distinction between two primary dimensions: self-efficacy (e.g., Bandura, 1997) and self-esteem (e.g., Orth & Robins, 2022). These dimensions correspond to what are sometimes referred to in social psychology as the “Big Two,” because they underly judgments of both ourselves and of others (Abele & Wojciszke, 2013). The self-efficacy dimension is commonly labeled strength, control, or competence, and I refer to it in the current study as agency. The esteem dimension is commonly labeled morality, moral-image, warmth, or communion, and I refer to it here as moral self-image.

Lastly, self-concept as a category of intermediate psychological change is useful for understanding how communication with the offender can make victims “feel better” (or indeed worse) because it is closely associated with overall well-being. A sense of agency is linked to well-being outcomes, as it determines people’s behavior (Bandura, 1997; Luszczynska et al., 2005), mediates the effects of daily stressors on mental health (Schönfeld et al., 2016), and can enhance recovery after traumatic events (Benight & Bandura, 2004), although its role in well-being may be more important in some cultures than others. The relationship between moral self-image (or self-esteem) and external measures of well-being may not be the simple positive linear correlation that it was once thought to be (Baumeister et al., 2003), but it does appear to be consistently associated with subjective measures of well-being, even in longitudinal studies (Orth & Robins, 2022). Moreover, victims thinking of themselves as a bad person because they were somehow morally responsible for a crime (what Hassija and Gray (2013) call “characterological self-blame”) is linked to the development and maintenance of trauma symptoms (Massad & Hulsey, 2006).

## Aim of this Study

Michelle wanted to know how meeting her father could make her “feel better,” especially when she said she had “got nearly 40 years of pain to deal with in an afternoon.” This study is an attempt to answer her question. More specifically, the research attempts to address three main questions: (1) does communication with the offender lead to changes in self-concept? (2) which components of victim communication with the offender lead to changes in victim self-concept? and (3) what types of changes in self-concept occur?

A qualitative study was the best way to elicit reflections of a sensitive nature in an ethical manner from a vulnerable, traumatized sample, enabling the participants to reflect on their own explanations for their experiences. Of course, with such data it is not possible to quantify, compare or eliminate relationships between variables of interest. Drawing on Ragin’s (1994) work, Maruna (2001) notes that “qualitative research is best suited for exploring similarity, not for establishing systematic differences” (p. 62). The current study accordingly draws on the expertise of victims to map “routes” from communication with the offender to changes in self-concept that are similar to routes identified by existing theories, or that are similar across participants.

## Method

### Participants

Semi-structured interviews were conducted in 36 cases, involving 40 direct and indirect victims. The 40 interviewees were aged between 16 and 73, the average age was 42 years. Two participants identified as Asian British, two as Black British, and the rest as White (36). Most of the participants identified as women (34 cisgender), six identified as men (5 cisgender and 1 transgender). Just under half of the participants (19) had been affected by sexual offences, ranging from a single incident of voyeurism to sustained childhood abuse. The others were directly or indirectly affected by burglary and theft (8), physical assault (8), harassment (1), slavery (1), fraud (1), manslaughter, and murder (2). Most of the victims knew the offender well (25), the rest said the offender was an acquaintance (6) or a stranger (9). All the crimes had been reported to the police except one, seven offenders had received a caution or community sentence, the other 28 offenders had served or were serving custodial sentences with a mean duration of approximately 6.5 years. The research took place between 5 months and over 40 years after the offence; with approximately a quarter of the offences having taken place in the past year, just over half having taken place within the past 3 years, three-quarters having taken place within the past 9 years and the final quarter having taken place 10 or more years prior to the interviews.

All participants had at least considered communicating with the offender. The extent of the communication that occurred ranged from none (e.g., in one case the victim only had a conversation with the facilitator about the possibility of communication with the offender) to multiple meetings between the victim and offender. In approximately three-quarters of the cases, contact was initiated by the victims. Some of these victims were certain they wanted to meet the offender and found out about the restorative justice organization as a means to do so, others were less sure but approached the organization to find out about their options. Under these circumstances, facilitators agreed to explore the possibility of communication with the offender, even though the offenders’ attitude toward the offence and the victim was not yet known. Facilitators explained to victims that the process was voluntary so it would only proceed if the offender also agreed, and that it would take time to prepare all parties. Standard procedure was for facilitators to also repeatedly check that victims wanted to proceed based on the information they had at each stage of the exploration/preparation process. In just under one-quarter of the cases, the victims responded to a more active offer of restorative justice—either because it had been initiated by the offender or because a criminal justice professional thought the case would be appropriate for referral.

Details of each case and the extent of their communication with the offender are found in Supplemental Materials. Interviews with victims before and after communication with the offender have been labeled Time 1 (T1) and Time 2 (T2), respectively.^3^ In all, 10 people were interviewed only at T1, 14 were interviewed only at T2, and 16 were interviewed at both time points.

### Recruitment and Data Collection

Two of the three restorative justice organizations I approached agreed to participate. A youth offending service agreed to participate but only referred one (adult) victim for the study, the rest of the participants were recruited through a charity delivering restorative justice with adult offenders, covering one police force area in South-East England. Facilitators were instructed by their manager to inform all victims about the research, unless there was reason to believe it would be inappropriate. The decision to offer participation to service-users was therefore left to facilitators’ discretion, and unfortunately no records were kept of the number of service-users who declined. No financial incentives were offered to participants.

In total, 57 people gave the facilitators verbal consent to participate in the research. Of these, 17 changed their minds or did not respond to contact after referral by the facilitator, resulting in the final sample size of 40. All 40 participants were provided with information about the study and the research team, and they gave written consent to participate before being interviewed. Consent from victims aged 16–17 was sought from both the direct victim and a parent/guardian, and both were considered participants in the study. The procedure for obtaining consent and other ethical considerations were approved in advance by Oxford University medical sciences research ethics committee. The interviews lasted on average 55 minutes, ranging between 15 minutes and 2 hours. Most were conducted in person, eight were conducted by telephone. The interviews were digitally recorded,^4^ transcribed, and analyzed using the data analysis software NVivo. Neither saturation nor a pre-determined sample size were used to limit the amount of data collection, rather the sample size was determined by collection of the maximum amount of data in the allotted timescale: April 2016–September 2018.

### Analysis

This study is part of a larger research project, whose aim was to understand the psychological changes expected and experienced by victims who communicate with offenders. Participants were asked broad questions about the crime and the offender, such as “In what ways has the crime affected you?” At T1, participants were asked about their desire to communicate with the offender, for example “How would you describe your expectations of the process?” At T2, they were asked questions about what had changed, especially regarding any expectations they had mentioned at T1. Initial analysis of the data led me to identify that victims expected and experienced change in their perceptions of the offender and the implementation of justice, which I discuss elsewhere (Batchelor, 2021) and changes in their self-concept, which are the changes I focus on in the current study.

I coded relevant parts of transcriptions and identified themes according to the six steps of thematic analysis outlined by Braun and Clarke (2006), having also conducted and transcribed all the interviews. I analyzed the data using thematic analysis, because as Braun and Clarke (2021) explain, “it can be used for a more deductive or more inductive analytic process” (p. 331) which was compatible with my two different but complementary goals. First, I sought to deductively assess whether victims described changes in self-concept corresponding to existing theories (such as those proposed by procedural justice theorists). Second, I sought to map the routes to change in self-concept described by victims that were insufficiently explained by existing theories. In practice, this means that I first assigned codes to statements in interviews that referred to any psychological change they expected or experienced (e.g., in emotion, cognition, or behavior) and any explanation victims gave for how or why this might occur. Where these codes aligned with existing theories of how self-concept might change through communication with the offender, I took what Braun and Clarke (2021) refer to as more “codebook” approach and collated those codes into themes predetermined by existing theory. Where victims’ descriptions of changes in self-concept were not previously described by theory, I took what Braun and Clarke refer to as “reflexive” thematic analysis approach. I developed codes into themes according to my interpretation of participants’ subjective experiences and the importance they assigned to them.

## Findings

Following the method of analysis outlined above, I grouped participants’ statements about how they thought the process had affected their self-concept into 10 themes, or routes to change. The findings section is organized by the components of the process that triggered these changes: (1) participation, (2) communication about the cause of the offence, and (3) communication about the consequences of the offence.

### Participation

#### Willingness to participate demonstrates agency and moral self-image

In this study, many victims mentioned that observing their own willingness to participate affected their self-concept, regardless of whether they were able to communicate with the offender. Several acknowledged that initiating the process was a risk, and that there were multiple ways it might make them feel worse rather than better (Willow, Sam, Casey, Hannah). Their choice to participate therefore constituted a direct demonstration to themselves and to others that they were strong, the kind of person who was willing to take risks. Casey, for example, said at T2:

I feel that I’m more confident within myself because I’ve gone through the necessary channels, and I’ve tried my best. Yeah, I would say that just knowing that I’m doing what I can, has helped to bring me out if that makes sense?

*Interviewer*: Because it’s something that you’ve decided to do yourself?

Yeah, because I’m just being proactive rather than just sitting on my hands, saying I want to do this, I want to do that, but never doing it.

Sometimes, the choice to participate appeared to constitute evidence that they were good people (positive moral self-image) rather than, or as well as, strong. Many victims described their decision to take part as an attempt to prevent reoffending, and that this was something they were proud of independently of whether it had been successful (Kaitlyn, Beatrice, Gemma, Kathy, Barbara, Mike, Dorothy, Oliver, Francis). Kaitlyn, for example, after the man who raped her declined to meet with her, said about him:

If he comes out and does it again to somebody else, I really have tried, to stop that. Even though it’s not really my responsibility. . . but, so I do feel like I’ve done everything I can to try and stop anyone else being in the same situation.

All the burglary victims interviewed at T1 suggested they chose to take part primarily to fulfil a duty to society (Mona, Rachel, Owen, Razik, Philip). Owen said he was “prepared to go along with it” because the facilitator thought it would be “worthwhile,” and Philip said, “if [the offender] is willing to go through the process, then it’s important that I go through it from the other side.” These findings align with previous studies suggesting that victims frequently state their motivation for participating is to “help” the offender (Coates et al., 2003; Doak & O’Mahony, 2006; Umbreit et al., 1994, 2001; Van Camp, 2017; Vanfraechem et al., 2009), and victims who participate tend to believe it makes the offender less likely to reoffend (Strang, 2002; Strang et al., 2006). Van Camp (2017) mentions as an aside that this may lead to victims seeing themselves as good people, noting that it is true of any altruistic act. Yet, the victims in this study suggest that observing their own willingness to make a sacrifice for the good of others played an important role in restoring their damaged sense of moral self-image, especially for the victims of serious and sexual crimes.

#### Participation demonstrates agency

Just as observing their own *willingness* to participate appeared to influence victims’ self-concept, victims observing their own actual participation in the process appeared to have a similar effect. Victims who took part in a meeting with the offender commonly described the meeting with the offender as having fulfilled their need to “face their fear,” “be brave,” “take a risk,” or get “over a hurdle” (Bridget, Kathy, Barbara, Zoe, Mike, Hannah, Michelle). Bridget said a new perception of herself as a brave person was the main outcome of the meeting, along with finding out that she had not been targeted:

I came away with peace of mind and the knowledge and the bravery, that I wanted to find, so yeah. I’m happy with the way it’s been left.

*Interviewer*: So it’s given you some peace of mind, partly from having the questions answered. . .?

Yep, and partly from being brave enough to do it. I’d say that was the main thing I think.

These comments suggest that some victims see the process as an exercise in desensitization, as suggested by Sherman et al. (2005). While this study cannot confirm Sherman and colleagues’ theory that exposure to the object of their fear (the offender) is the mechanism by which victims become less fearful, it suggests that some victims believe it to be. Victims mentioned that observing themselves facing such a significant object of fear demonstrated their own agency and courage.

#### Participation enables reevaluation of offender’s relative strength

For some victims, a meeting with the offender replaced their fear of the offender with an increased sense of agency relative to the offender. Mike, for example, commented on his own sense of relative power when he met with the man who had kept him in forced labor for many years:

When I used to see him, or he used to make us do things, he had the power. For someone to have no power is a very different way of looking at things.

*Interviewer*: How was that for you then?

I felt quite happy. Because, it was always that he had the power, and the threats. So instead of being a big man, he was like a little man.

Hannah also said she derived confidence from seeing that the man who had abused her looked nervous and small. Oliver said that before the meeting he had been worried about what would happen if he met in the street the man who had stabbed him multiple times, but this was rectified by the discovery that the offender was not as strong as he remembered, “Last time I see him he was kind of hench. But when I see him, he had walking sticks, he had all these bone problems.” Previous literature suggests that communicating with the offender often results in a change in victim perceptions of the offender, usually from “monster” to less “evil” or less powerful (Aertsen & Peters, 1998; Beven et al., 2005; Hoyle et al., 2002; Strang et al., 2006; Umbreit & Vos, 2000; Umbreit et al., 1994; Walters, 2015). This study suggests that such a change is also linked to a change in victims’ perceptions of themselves.

#### Procedural justice signals victim is valued

The participants frequently mentioned that having or attempting contact with the offender through the restorative justice service gave them a sense of having a voice, feeling listened to, being respected and supported, getting information, and generally having some degree of control over the process. Several also explicitly suggested that this contributed to their overall sense of agency. Terri’s father, the perpetrator of childhood sexual abuse, was denying the offence but was willing to meet with her, so Terri felt in the end that she had agency in being able to exercise her choice not to meet with him:

So [an RJ process is] an option open for the rest of my life if I wanted it. And, it’s nice that I feel that I had control of all that. That I had somebody who respected the way I felt, and didn’t tell me I was stupid.

These findings align with previous research, supporting the theory that experiencing procedural justice as part of communication with the offender, can lead to victims feeling an increase in agency and feel valued. This has been documented in detail elsewhere so I shall not describe it any further here (Beven et al., 2005; Miller & Hefner, 2015; Strang, 2002; Van Camp & Wemmers, 2013; Wemmers & Cyr, 2005, 2006).

### Communication About Causes of the Offence

#### Offender non-participation communicates offender’s relative moral image

Many victims initially expressed feelings of guilt and shame, either about the offence or its consequences for the offender. Some of these victims subsequently interpreted the offender’s nonparticipation or lack of remorse as confirmation that the crime was in fact the offender’s fault, that the punishment was deserved, and that their own sense of self-blame was misplaced (Terri, Willow, Kaitlyn, Rose, Casey). Willow, a young teenager, said at T1 that she wanted to know why her boyfriend had raped her, as she worried that she was partially to blame. At T2 her mother said:

[Willow] has actually started to see that now I think, that really it wasn’t her fault. And that he was the older person - this has developed in the last few months in her head - since he said no [to meeting with her]. . . . She said “oh he’s not very nice is he? And he is a liar and, he’s this that,” whereas in her head beforehand it was all “he’s a nice person and maybe it was me.”

Casey was sexually abused by a family friend at a young age and reported it many years later. She said at T1 she knew it was “irrational” to blame herself for his prison sentence, but that was still just “how I’m feeling.” At T2, after he declined to meet her, she said:

I did struggle a lot to begin with, him being in prison, I was blaming myself and things like that. Where, to an extent I still do. But, not as much. Because he hasn’t done a, b, and c to at least try to prove that he is remorseful.

#### Acknowledgment and apology communicates that the victim is not weak or to blame

Some victims said they appreciated acknowledgment of the crime because it alleviated their doubts about whether the crime happened as they remembered, making them feel more in control. Faye, who was sexually abused by her older brother, said “I wanted to know, was it everywhere we lived? Was it over that 10, 11, 12-year period? He said, ‘yes it was’. So it wasn’t my imagination.” Others felt more in control because the offender expressed remorse, suggesting they would not target the victim again. Carol, for example, said “I was no longer frightened of meeting him. We’d had our meeting, we’d cleared the air. He’d apologized profusely, we believed him.”

Some victims felt guilt or shame because of the offence or its consequences, and hearing the offender take responsibility helped to restore their moral self-image. Barbara had felt guilty about calling the police when her son assaulted her, but having met him, she said “The relief of the guilt lifted, you know and ‘no it wasn’t me, it was you, because I heard you say it.’” Naomi received a letter from the offender, an ex-boyfriend who had distributed indecent images of her, although she was ultimately unable to meet with him. She said that in the letter, “he apologized that he was wrong. And it finally kind of shut it down, that I was believed. And I wasn’t the one in the wrong, he was the one in the wrong.”

We have long known that acknowledgment and apology from the offender are important to victims (Tavuchis, 1991), and some have theorized that this is primarily because it restores the victim’s sense of agency (Shnabel & Nadler, 2008, 2015). This study has shown that acknowledgment and apology can also be a route to an enhanced sense of moral self-image for the victim.

#### Learning the choice of victim was random communicates the victim was not weak or to blame

In this study, two victims said that learning they were not personally targeted helped restore a sense of agency or moral self-image. Bridget was relieved to hear from the man who raped her that she was not targeted because she was vulnerable, increasing her sense of agency: “I was under the impression that I was stalked, but no I was just in the wrong time at the wrong place.” Zoe was relieved to hear that she had not provoked her friend’s violent attack against her, and that she was not to blame. She said meeting with him made her feel better about herself “because I know it was nothing that I did, it could have been anybody.”

Victims often ask “why me?” (Maguire, 1980), and some wish to ask this question of the offender (Doak & O’Mahony, 2006). This study confirms existing proposals that when offenders respond that the victim was not personally targeted—that the choice of victim was random—this can provide victims with a sense of relief (Sherman et al., 2005; Strang et al., 2006). However, many victims received answers to “why me?” that they disbelieved or found unsatisfactory, and their response is discussed in the next section.

#### Challenging the offender’s reasons demonstrates agency and moral self-image

Some victims said they believed they *had* been specifically targeted but wanted to meet the offender anyway. Razik said he thought his house had been chosen because burglars “target Asian houses,” and Francis said he was violently attacked because of his identity as a transgender man. They were not willing to accept (and were certainly not reassured by) offender attempts to deny that they were personally targeted. Rather, they saw the meeting as a chance to challenge the offenders’ biases. Francis said that the offender had “tried to pass the buck” in the meeting, but Francis said to him “‘I’m gonna cut you off dead’, it was me telling him that he didn’t have the power to win, he wasn’t telling the truth.”

Other victims also appeared to affirm their sense of agency and/or moral self-image by voicing their disbelief or challenging the offender’s narrative about the offence (Denise, Lisa, Karen, Nita, Gemma, Philip, Mike, Dorothy, Hannah). Hannah, who was sexually abused as a child by her mother’s partner, received a letter from the offender with “reasons” about why he committed the offence:

I needed a letter to try and gauge how I was going to feel. And got that. And actually was laughing at the letter cos it was so pathetic.

*Interviewer*: Pathetic in what sense?

He was trying to make excuses, he was blaming it on drugs.

She then went on to meet with him, and during the meeting she said she had repeatedly challenged the stories he told about his offences:

He kept calling it “dysfunctional behavior.” So, and I said to him, “that’s something you’ve learnt in some therapy thing, you’re not acknowledging what you’ve done. You’ve abused a child . . . just say those words.”

Observing oneself not only “facing” the person who was the object of their fear, but also challenging and confronting them, appeared to give these victims a sense of control over the situation and restore their sense of moral self-image almost as much—or even more than—hearing an apology or an exculpatory reason for the offence.

### Communication About Consequences of the Offence

#### Learning about consequences for the offender increases self-worth and reduces self-blame

Several victims said that learning the offender’s punishment was proportionate to the offence helped them feel valued by society (if they had previously thought the punishment was too lenient), less guilty (if they had worried the punishment was excessive), or sometimes both. Seeing the offender in prison or hearing about the nature of their community sentence made them feel they had been valued because the crime had been taken seriously (Gemma, Hannah, Karen, Mike, Oliver). Bridget said specifically that hearing about the criminal justice and personal consequences for the offender had given her a bit of “power back.” Those who felt guilty about calling the police or felt responsible for the offender’s sentence benefited from hearing that the offender acknowledged that the sentence was deserved or even helpful (Sadie, Karen, Kathy, Barbara, Zoe). Kathy, for example, who was attacked by a friend, said that before the meeting she felt “a bit of a failure” because the situation led her to calling the police. However, she said that hearing him say his sentence had “given him time out, to. . . reflect and clean himself up” she had been reassured that calling the police “was the right thing to do.” In Karen’s meeting with the young man who stole from her, she even dealt with her guilt about calling the police by explaining and apologizing for her actions:

Restorative justice gave me a chance to face up to him and say why. And give him reasons for my behavior, for saying “sorry you’re going to be arrested here and taken away.”

Victims appreciate hearing about offenders’ responses to their punishment (Funk et al., 2014) and in restorative justice processes this can form part of how victims benefit (Batchelor, 2021). This study points to the specific role of discussing the consequences for the offender as a route to change in victims’ self-concept.

#### Describing impact and recovery creates distance between consequences of offence and self-concept

For most of the victims in this study, describing the impact of the crime to the offender appeared to be a means of distancing themselves and their character from the emotions, thoughts and behaviors that were caused by the crime. Victims saw it as an opportunity to talk about their fear or anger, for example, but to also highlight that those emotions were caused by the offence and not by the victim’s own weakness of character. In addition, when victims were able to talk about the ways they had overcome the suffering they experienced, this further demonstrated their strength. Sam said that she wanted her father to know the impact his sexual abuse had on her because “it’s not fair for him to think that everything is hunky dory and everyone can forget what he done.” She went on to say:

And that is part of why I want to do it. Because I want to show him that despite what he’s done I have come out the other side. I am still standing.

Hannah said she had developed coping strategies over the many years since the offence, and that demonstrating this new self to the offender was an important part of the process for her:

To be able to go and tell him that. . . I don’t consider myself a victim, cos I’m not. I haven’t let it beat me. I did for a bit. But, I call myself a survivor. So cheesy, but. . .

Some victims were more concerned with regaining their moral self-image because they felt the effects of the offence had led them to live a life that others judged to be bad. Faye wanted to take part to demonstrate to her family that some elements of her personality and lifestyle were not evidence of a bad character, but were in fact a result of the abuse:

I need to justify how I am, because of what his actions, what he did, and it was over a long-term. So it has affected me different ways, or what-have-you. Which, I’ve been shunned by my family because of it.

Many have documented victims’ desires to convey the impact of the crime to the offender (Bolívar et al., 2009; Borton, 2009; Coates et al., 2003; Umbreit & Coates, 1992; Umbreit & Vos, 2000; Umbreit et al., 2001), and proposed reasons victims may want to do so, such as preventing the offender from reoffending (Van Camp, 2017), or simply to express emotions (Rossner, 2011). Bolívar (2019) suggests that it is a two-stage process, in which victims first have their victim status recognized *and then* they can go on to rebuild their identity. However, this study suggests that differentiating between the victim’s character and the impact of the crime by discussing it with the offender can itself be a route to increasing the victim’s sense of agency and moral self-image.

### Negative and Absent Change

The participants tended to describe the changes in self-concept that they experienced in positive terms, and I have mapped them above on the basis that victims had insight into their own experiences, which they provided access to through interviews. It was not possible in this study to quantify negative outcomes or to compare experiences in the presence and absence of each component. In addition, the participants who experienced the most serious negative outcomes may be those who withdrew from the study. However, given the problem that restorative justice research often neglects to investigate potential negative effects, I briefly consider whether there was evidence that the changes described by most victims as positive could also have negative effects, and consider what victims said about their absence.

The current study does not include the perception of offenders, so it is impossible to assess whether the processes described by victims in this study had any negative effects on the offenders. However, as pointed out by Delgado (2000), there is a particular danger of negative effects for the offender where there is a power balance in favor of the victim. While it is not something this study had the capacity to assess directly, as a researcher I noted that two routes to change in victim self-concept appeared to have particular *potential* for a negative impact on the offender when the victim was relatively powerful: powerful victims participating to feel “good” about themselves (increase moral self-image), and powerful victims attempting to feel even more powerful through challenging the offender’s narrative about the offence. Achievement of any victim goals must therefore be weighed with the needs and well-being of the offender.

Some changes experienced as positive by the victim could have negative effects of which they were unaware or over the long term. One concern raised by Walters (2015), for example, is that victims who reevaluate the offender may then feel guilty for “liking” them. Philip, in this study, said that for 24 hours after his meeting with the offender he felt *more* vulnerable, because he had given the offender personal details. While for Philip this effect receded over time, it raises the possibility that communication with the offender may lead to a reduced rather than increased sense of agency for some. It is worth noting, therefore, that the absence of many negative effects could occur in part because this study was unable to capture longer term or more subtle negative effects.

Although offender nonparticipation led to positive change in self-concept for some, many victims in the current study suggested that it also had negative effects. The five victims I was able to interview about their experiences of offender nonparticipation interpreted it such that they could derive strength and a moral self-image, but also noted they were then unable to access other components of the process that could have been beneficial, namely, communication with the offender about the causes or consequences of the offence. Five other victims declined a T2 interview after the process was halted either because the offender had reoffended (Mona) or the offender declined to meet with them (Marie, Sam, Emma, Mona, Naomi). It is impossible to know, therefore, what negative effects these victims might have experienced as a result of the process.

A glimpse into the most concerning negative effects of offender nonparticipation is provided by the victims in this study who interpreted it as an absence of procedural justice. While a process that is voluntary for *both* offender and victim might *technically* be fair, victims who wished to communicate with the offender interpreted this arrangement to mean that they had less control over the process than the offender. Although Kathy’s meeting with the offender eventually went ahead, it was delayed several times because the offender changed his mind about participating, and she commented that whether the meeting would take place appeared to be “down to him more than me.” Gemma, whose meeting with the offender also went ahead, nevertheless noted that “he had all of the control really. Because he could have just said no, and then it would have been end of.” Unsurprisingly, those who wanted to but were unable to meet with the offender felt this lack of process control most acutely. Rose, Kaitlyn, Lisa, and Willow were all disappointed that the offender had the “last word” about whether they would be able to meet. Therefore, while a novel contribution of this study is that offender nonparticipation can *sometimes* result in positive psychological change for victims, the possibility remains that offender nonparticipation is damaging for other victims.

Even when the offender did participate in the process, not all victims experienced all the components that might have benefited them. Some victims described these absences as unimportant, for example many said that they did not want or need an apology. Some victims were even able to turn an absence into a personal benefit, for example, where they felt the offender’s given reason for an offence was unacceptable, they could observe themselves challenging the reason, as described above. However, for others these absences were clearly a source of disappointment. Several victims mentioned that because the offender had been unable or unwilling to explain why they committed the offence, the victim continued to ruminate on whether they were responsible for triggering the offence in some way (Michelle, Lisa, Nita). When victims remained dissatisfied with the offender’s sentence (Nita) or felt that justice had not been fully achieved (Faye), communication about consequences of the offence was not a viable route to positive changes in victim self-concept. This is an especially notable absence, as low rates of reporting and conviction mean that in practice most offenders would not have any consequences to discuss—at least none that had been externally imposed—and that this route is effectively inaccessible to many victims.

The potential impact of an absence of expected components is illustrated by Nita’s dissatisfaction after her meeting with the man who took indecent photos of her without consent. The source of Nita’s dissatisfaction is hard to isolate, as she described an absence of so many components; saying the offender could not explain why it happened, and he did not acknowledge that his behavior was wrong or that it had an impact on her. She thought his sentence was too lenient, and that he interrupted and spoke over her in the meeting (so she did not experience the process to be just). Nita was unclear about whether these absences were damaging (negative) or merely disappointing (relatively neutral), saying at one point she felt she was “violated a second time by somebody who really isn’t that sorry” but summarizing the experience as just a “waste of time” because it did not make her “feel better.” Either way, it is instructive that the absence of the components in this study, especially when expected by victims, may lead to disappointment and even have the potential to damage rather than enhance a victim’s self-concept. The lack of data from victims who have negative experiences remains a pervasive and concerning obstacle in research on restorative justice schemes, one which this study has only been able to surmount to a small extent.

## Discussion

The 10 routes to change in self-concept described by participants in this study are set out in Table 1. Gecas (1982) outlines four “sources” of self-concept which I employ here to discuss the ways in which the individual components of a restorative justice process could lead to the changes participants described: reflected appraisal, social comparison, self-perception, and changes in psychological centrality.

**Table 1.** Routes to Change in Self-Concept.

Route	Existing Literature Summary	Source of Change
Participation		
Willingness to participate demonstrates agency and moral self-image	Previous explanations focus on “facing fear” but effect of *willingness* to participate unexplored	Self-perception
Participation demonstrates agency	“Desensitization” theories may partially explain this route, but links between self-perception and changes in self-concept not previously explicit	Self-perception
Participation enables reevaluation of offender’s relative strength	There is a documented route to change in perception of *offender*, but links to victim self-concept previously unexplored	Social comparison
Procedural justice signals victim is valued	Well-documented route from participation to change in self-concept	Reflected appraisal
Communication about causes		
Offender non-participation communicates offender’s relative moral image	To date unexplored	Social comparison
Apology and acknowledgment communicates the victim is not weak or to blame	Apology and acknowledgment known to be important and can restore sense of agency, but links to moral self-image unexplored	Reflected appraisal
Learning the choice of victim was random communicates they are not weak or to blame	Commonly thought to be reduce self-blame but evidence for effect of communication with the offender on self-blame is mixed	Reflected appraisal
Challenging the offender’s reasons demonstrates agency and moral self-image	To date unexplored	Self-perception
Communication about consequences		
Learning about consequences for the offender increases self-worth and reduces self-blame	Importance of punishment to victims in restorative justice processes known, but link to self-concept previously unexplored	Reflected appraisal
Describing impact and recovery creates distance between consequences of offence and self-concept	Known that victims often wish to describe impact, and some theorization about link to self-concept, but nature of link previously unclear	Psychological centrality

Reflected appraisal means that people’s view of themselves derives partially from how the person believes others see them. In this study, there were numerous examples of victims receiving a message about their self-concept from an external source—whether through the process, the facilitator, or the offender. Experiencing procedural justice communicated that the victim was valued. Acknowledgment and apology communicated that the victim did not imagine the crime (restoring a sense of agency) or that they did not cause the crime (restoring a sense of moral self-image). Hearing that they were not personally targeted communicated that the victim was not vulnerable or to blame, and discussing the consequences for the offender communicated both that the victim was valued and that they were not a bad person.

Gecas explains that a person’s view of themselves is also shaped by social comparison; how they view themselves in comparison to others. In this study, victims who initially perceived the offender to be powerful because they had control at the time of the crime, reevaluated the relative strength of the offender through meeting them, and thus felt themselves to be stronger. Some victims also interpreted the offender’s unwillingness to meet as a sign that the offender was either weak or bad, which reduced their own self-blame and made them feel relatively strong and good in comparison.

Self-concept can also derive from observing ones’ own behavior, or what Gecas calls self-perception. In this study, victims observed their own willingness to take part, as well as their own participation, and used these observations to determine that they must be brave and/or good people. When victims observed themselves challenging the offender, this also enhanced their sense of agency.

Gecas lastly posited that the substance of our self-concept depends on the psychological centrality of its various aspects. In this study, communicating with the offender led to a change in the psychological centrality of the consequences of the offence. Victims acknowledged that they had felt weak or bad after the crime, but emphasized that this was a result of the offence rather than a core aspect of their character. Describing their recovery also enabled them to focus on a core self that was good and strong.

The multiple routes to change in self-concept mapped in Table 1 shed light on what some people mean when they say that communication with the offender enables them to feel less like a victim (e.g., victim quoted by Umbreit et al., 2001). Research on trauma tells us that “the core experiences of psychological trauma are disempowerment and disconnection from others” (Herman, 2015, p. 133). Victimization is a direct violation of a person’s own agency; thus, it increases perceptions of the likelihood and impact of future victimization, and decreases the extent to which people believe they will have control in the future (Jackson & Gouseti, 2016). Crime also often affects victims’ moral self-image, both because the nature of the crime can cause shame and because humans want to believe that the world is just, so if something bad happens to someone we are inclined to believe they are a bad person, even when that “someone” is ourselves (Lerner, 1980). Regaining a lost sense of agency or moral self-image through one of the ten routes described here, is thereby a means of discarding a victim identity.

Returning to Michelle’s dilemma in the introduction, a map of routes to change in self-concept can help other victims in her position to make an informed decision about whether to communicate with the offender, to recognize and articulate their own goals, and to identify possible ways of fulfilling them (including but not limited to communication with the offender). A fully fleshed out map, to which this study contributes one part, could also help facilitators to better prepare victims and help them manage expectations. For example, if risk-taking is indeed an important route to increasing victims’ sense of agency, it is important that facilitators do not suggest they can eliminate the risk involved in meeting the offender. Practitioners and support services should make victims aware that they cannot fully control the process, and they may find themselves upset if, for example, the offender chooses not to participate. However, they can also draw on the findings here to help victims interpret offender nonparticipation such that it nevertheless increases the victim’s sense of agency and moral self-image.

### Limitations

The current study maps only a subset of psychological changes experienced by victims communicating with offender; there are many types of psychological change that victims experience beyond changes in self-concept, and there are doubtless many other routes to change in self-concept that the victims in this study did not identify. The interview schedule did not include specific questions about self-concept, as this theme was developed in response to the data, so this is an avenue for future studies which could collect specific measures of self-concept before and after communication with the offender.

The participants in this study all live in South-East England, and the majority identified as White British, cisgender women (for more detail see Method: Participants). Just as with any small sample, therefore, this research should not be used to generalize to a broader population of victims, nor does it allow for comparison between groups, for example by gender or crime type. However, the purpose here was to map *potential* routes to changes in self-concept, and the victims who participated in this study contributed a range of such routes, based on diverse experiences of victimization and of communication with the offender. I have created a flexible model because future research is likely to identify additional routes to change in victim self-concept through communication with the offender. When people who were underrepresented in the current study describe routes to change that I have not documented, these can be added to the model.

In this study, victims’ responses may have been influenced by their beliefs about the expectations of the researcher (Lumsden & Winter, 2014), and by the expectations of the restorative justice facilitators who referred them for the research. Despite all efforts to convey to participants that the research was independent from the restorative justice service, it was clear that some victims did not make a distinction. Almost all the victims in this study chose not to have any further contact after the final interview because they said they wished to put the offence behind them, so I was unable to return to any of the participants for their reflections on these findings. It was also beyond the scope of this victim-focused article to assess how victims’ expectations and experiences affected the offenders with whom they communicated.

## Concluding Remarks

In a common version of a script for facilitators of meetings between victims and offenders, the facilitator says to everyone present, “We will focus on what [offender name] did and how that unacceptable behavior has affected others. We are not here to decide whether [offender name] is good or bad” (e.g., IIRP, 2010). This is in accordance with a commonly cited underlying principle of restorative justice; separation of the crime from the person (Braithwaite, 1989), or the “deed from the doer” (Wachtel, 1999). Like many offenders, the victims in this study felt that their self-concept had been defined and sometimes destroyed by the crime. Communication with the offender helped to redefine themselves as morally good people with agency, and to distance themselves from the label of rather than merely a victim. Thus, while a primary stated goal of restorative approaches may be to separate “the deed from the doer,” victim–offender communication can also play a significant role in separating “the deed from the done-to.”

## Supplemental Material

sj-docx-1-jiv-10.1177_08862605221119725 – Supplemental material for Separating the “Deed” From the “Done-To”: How Communicating With the Offender Can Change Victims’ Self-Concept Click here for additional data file. Supplemental material, sj-docx-1-jiv-10.1177_08862605221119725 for Separating the “Deed” From the “Done-To”: How Communicating With the Offender Can Change Victims’ Self-Concept by Diana Batchelor in Journal of Interpersonal Violence

## Notes

1. All participant names are pseudonyms.

2. The multiple definitions and history of restorative justice have been described by numerous authors, summarized by Daly (2016).

3. Where some communication with the offender had taken place but the process was ongoing, these interviews have also been labeled T1. This is because the participants themselves spoke primarily about their expectations of communication with the offender, as if it had not yet occurred (e.g., some of these interviews took place after an exchange of letters but before a meeting with the offender, or after informal contact with the offender but before an official meeting with a facilitator present).

4. All except one participant agreed to recording. Razik gave his lack of fluency in English as his reason for declining.
