# Supplementary material for: Separating the “Deed” From the “Done-To”: How Communicating With the Offender Can Change Victims’ Self-Concept
Source: J Interpers Violence. 2022 Sep 4;38(5-6):4877–905. doi: 10.1177/08862605221119725 (PMC9900692; doi:10.1177/08862605221119725)
Supplement: sj-docx-1-jiv-10.1177_08862605221119725 – Supplemental material for Separating the “Deed” From the “Done-To”: How Communicating With the Offender Can Change Victims’ Self-Concept [file sj-docx-1-jiv-10.1177_08862605221119725.docx]

| *Supplementary Material*  Details of 36 cases (40 people)* | | | | | | |
| --- | --- | --- | --- | --- | --- | --- |
| # | Pseudonym | Interviews | Crime | CJS Response | Offender relationship to victim | RJ |
| 01 | Lydia | T1 | Burglary | Custody: 4yrs | Acquaintance | None |
| 02 | Terri | T2 | Rape | Custody: 18yrs | Father | None |
| 03 | Megan | T1 | Sexual assault | Custody: 10yrs | Acquaintance | None |
| 04a | Willow | T1 | Rape | Custody: 5yrs | Boyfriend (online) | None |
| 04b | Imogen (Willow’s mother) | T1&2 | Rape of daughter | As above | Boyfriend of daughter | None |
| 05a | Marie | T1 | Rape | Custody: 12 mths | Boyfriend | None |
| 05b | Linda (Marie's mother) | T1 | Rape of daughter | As above | Boyfriend of daughter | None |
| 06 | Sam | T1 | Rape | Custody: 19 yrs | Stepfather | None |
| 07 | Kaitlyn | T1&2 | Rape | Custody: 6 yrs | Friend/acquaintance | None |
| 08 | Emma | T1 | Domestic fraud | Custody: 2yrs | Partner | None |
| 09 | Mona | T1 | Burglary | Custody: 6 mths | None | None |
| 10 | Rose | T1&2&3 | Sexual assault | Custody: 6 yrs | Client | Letter sent |
| 11 | Casey | T1&2 | Sexual assault on a child | Custody: 4 yrs | Family friend | Letter sent |
| 12 | Denise | T2 | Harassment | Youth Conditional Caution | Friend of daughter's | Letter rcvd |
| 13 | Lisa | T1&2 | GBH | Custody: 2 yrs | Partner | Letter rcvd |
| 14a | Sadie | T1&2 | Rape (of sister and mother) | Custody: 19 yrs | Father | Letter rcvd |
| 14b | Abby (Sadie’s mother) | T1&2 | Rape | As above | Ex-husband | Letter rcvd (by daughter) |
| 15 | Naomi | T1 | Sexual assault on a child | Custody: 10 years | Boyfriend | Letters sent & rcvd |
| 16 | Beatrice | T2 | Class A Possession (led to victim daughter’s death) | Community Sentence | None | Letters sent & rcvd |
| 17 | Tasha | T2 | Assault (physical) | Community Sentence | Friend | Conference |
| 18 | Karen | T2 | Theft | Custody: 12 mths | Lodger | Conference |
| 19 | Bridget | T2 | Rape | Custody: 8 yrs | None | Conference |
| 20 | Nita | T1&2 | Voyeurism | Community Sentence | None | Conference |
| 21 | Carol | T2 | Burglary | Community Sentence | Friend/acquaintance | Conference |
| 22a | Rachel | T1&2 | Burglary | Custody: 4 yrs | None | Conference |
| 22b | Owen | T1&2 | Burglary | As above | None | Conference |
| 23 | Gemma | T2 | GBH & Robbery | Custody: 4 yrs | Acquaintance | Conference |
| 24 | Kathy | T1&2 | ABH | Custody: 3 yrs | Friend | Conference |
| 25 | Razik | T1 | Burglary | Custody: 10 mths | None | Conference |
| 26 | Barbara | T2 | Threats to kill | Custody: 2 yrs (IPP) | Son | Conference |
| 27 | Zoe | T2 | GBH/ Wounding (S.18) | Custody: 6 yrs | Friend | Conference |
| 28 | Philip | T1&2 | Burglary | Custody: 3 yrs | None | Conference |
| 29 | Faye | T1&2 | Rape | Not reported | Brother | Conference |
| 30 | Mike | T2 | Slavery & threats | Custody: 6 yrs | Acquaintance | Conference |
| 31 | Dorothy | T1&2 | Murder | Custody: 30 yrs | None (known to son) | Conference |
| 32 | Hannah | T2&3 | Rape/child abuse | Custody: ?yrs (IPP) | Stepfather | Conference |
| 33 | Brenda | T2 | Sexual assault on a child | Caution | Stepson | Conference |
| 34 | Oliver | T2 | Wounding (S.18) | Custody: 3 yrs | Acquaintance | Conference |
| 35 | Michelle | T1&2 | Sexual Assault on a child | Custody: 3 yrs | Father | Conference x2 |
| 36 | Francis | T1&2 | Assault/robbery | Custody: 2 yrs | Acquaintance | Conference x2 |
| **All information about cases provided by victims and could not be independently verified. Custody length given in months if under 1 year, else to the nearest year.* | | | | | | |
